# Preparing for Ageing: A Cross-Sectional Pilot Study at a Tertiary Care Hospital in Kannur, India

**DOI:** 10.7759/cureus.94580

**Published:** 2025-10-14

**Authors:** Anand Anilkumar, Mohammed Haneef Aloodigothi

**Affiliations:** 1 Internal Medicine, Aster Malabar Institute of Medical Sciences (MIMS), Kannur, IND

**Keywords:** ageing preparedness, cross-sectional pilot study, elderly, financial security, geriatric health, kerala, mental preparedness, retirement planning, social support

## Abstract

Background: In India, the ageing population is expanding steadily, with noticeable regional variations in the proportion of older adults. Although Kerala has high literacy and health indices, gaps in financial and legal readiness persist. As this is a hospital-based pilot study, findings are exploratory and not generalisable. Assessing preparedness for ageing is essential in low- and middle-income countries like India, especially in states with longer life expectancies such as Kerala.

Objective: This study aimed to assess the level of mental preparedness for ageing among the elderly population in Kannur, Kerala, and to identify key demographic, health, and financial predictors influencing it. The novelty of this work lies in its focus on “mental preparedness”, a dimension rarely studied in India.

Methods: A hospital-based pilot cross-sectional study was conducted at a tertiary care facility in Kannur, Kerala. A structured questionnaire (self-developed and underwent expert validation) was administered to 305 elderly individuals recruited by purposive sampling. Data were analysed utilising descriptive statistics, chi-square tests, and multivariate ordinal logistic regression to explore associations and predictors of mental preparedness for ageing. Given the cross-sectional design, associations rather than causal relationships are emphasised.

Results: Approximately 68% of participants felt mentally prepared for ageing. Bivariate analysis revealed significant associations with education, marital status, occupation, health status, financial planning, and social support (p < 0.05). Multivariate analysis confirmed that perceived financial security (adjusted odds ratio (AOR) = 4.08), presence of a pension plan (AOR = 2.24), having health insurance (AOR = 0.36), and strong social support systems (AOR = 7.56) were independently associated with higher mental preparedness. The model demonstrated good fit (χ² = 63.89, p < 0.0001; c = 0.736).

Conclusion: Mental preparedness for ageing in this pilot hospital-based study was found to be associated with financial stability, pension coverage, health insurance, and social support. However, the exploratory nature of the study, purposive sampling, use of a single, non-validated item, and unmeasured confounders limit the generalisability of the findings. The study’s novelty lies in highlighting mental preparedness, a rarely studied dimension in India, and its multidomain perspective. These preliminary results point toward the importance of financial and social interventions to enhance preparedness, but larger community-based, longitudinal studies using validated tools are needed to confirm these associations and guide policy.

## Introduction

India's demographic landscape is continually going through a significant shift, with the percentage of elderly individuals projected to rise from 10.1% in 2021 to more than 20% by 2050 [1]. Known for its high literacy and life expectancy, Kerala is leading this transition [2]. Ageing is often accompanied by increased dependency, chronic illness, and emotional vulnerability. While physical and medical aspects of ageing have been widely studied, mental preparedness is still not fully explored [3,4]. Research suggests that individuals who plan financially, socially, and psychologically for old age tend to have better outcomes in later life [5-7].

Despite Kerala’s strong social and health indicators, previous reports highlight persisting gaps in financial literacy, pension awareness, and legal preparedness among the elderly [8,9]. This paradox makes Kerala an important setting to examine “mental preparedness” for ageing.

The novelty of this study lies in its focus on mental preparedness, a multidimensional construct encompassing perceptions of readiness across health, financial, social, and legal domains, which is rarely studied in India. Most existing studies are descriptive and neglect psychological and planning-related aspects. By addressing this gap, the study contributes early pilot evidence from a state with the most advanced ageing profile in the country.

The objectives of this study were (i) to estimate the prevalence of mental preparedness for ageing among older adults in Kannur, Kerala, (ii) to examine the socio-demographic, health, financial, and social correlates of preparedness, and (iii) to identify the independent predictors influencing preparedness for ageing.

## Materials and methods

Study design and setting

This was a descriptive cross-sectional pilot study conducted over a three-month period (March-May 2025) at Aster Malabar Institute of Medical Sciences (MIMS), a tertiary care hospital in Kannur district, Kerala, India. The hospital caters to a substantial semi-urban and rural community and sees a considerable number of elderly patients in outpatient care, making it a suitable setting to study ageing preparedness. As this is a hospital-based purposive sample, findings are not generalisable to the community.

Participants and sampling

Participants were elderly individuals aged 60 years and above attending the hospital during the study period. Purposive sampling was used to recruit 305 participants. The eligibility criteria encompassed individuals who were 60 years of age or older, mentally competent to answer survey questions, and willing to provide informed consent. Patients with severe cognitive impairment or acute illness at the time of the interview were excluded. Because this was a pilot study, no formal sample size calculation was performed, which is acknowledged as a limitation.

Data collection tool and procedure

Data were collected using a structured questionnaire tailored specifically for this study (Appendix A). The tool consisted of 23 items covering five domains: demographics, health status, financial preparedness, mental and emotional well-being, and future planning. The questionnaire was developed after reviewing existing tools (e.g., Longitudinal Aging Study in India (LASI), World Health Organization Quality of Life-AGE (WHOQOL-AGE), Preparation for Future Care Needs Scale) and subsequently underwent expert validation, involving review by a geriatrician and public health expert for face and content validity. However, it was not subjected to reliability testing or psychometric validation, which limits interpretability. Data were collected by trained personnel in outpatient and inpatient settings to ensure consistency. Steps such as standardised training and neutral wording were used to reduce recall and social desirability bias, but these could not be eliminated. Although data collection was conducted by trained personnel with standardised instructions, no formal inter-rater reliability testing was performed. This represents a potential source of variability in questionnaire administration.

Statistical analysis

Data were entered and analysed using IBM SPSS Statistics software, version 28 (IBM Corp., Armonk, NY). Descriptive statistics (frequencies, percentages, mean ± standard deviation) summarised participant characteristics and preparedness indicators. Chi-square tests were performed to evaluate the associations between mental preparedness and socio-demographic or financial factors. Variables significant at p < 0.05 in bivariate analysis were incorporated into a multivariate ordinal logistic regression model to determine independent predictors of mental preparedness. Multicollinearity was assessed using the variance inflation factor (VIF < 2). Model fit was evaluated (χ² = 63.89, p < 0.0001; c = 0.736), indicating good discrimination. Statistical significance was set at p < 0.05.

## Results

A total of 305 participants aged 60 years and older were included. The sample was balanced by gender (50.5% male), with most in the 60-69 age group (46.2%). About one-fifth had no formal education, while one-third had completed secondary school. The majority were married (76.4%) and from middle socio-economic backgrounds (64.3%). Most participants reported at least one chronic illness (83.6%), though two-thirds underwent annual health check-ups. Financial indicators showed that while 70.8% were satisfied with their savings, only 35.1% had extensively planned for retirement. About 64% received a pension, and 56% had health insurance. Legal preparedness was lower, with only 44.6% having wills or power of attorney (Table 1).

**Table 1:** Descriptive characteristics of the study population (N = 305)

Characteristic	Category	n (%)
Gender	Male	154 (50.5)
	Female	151 (49.5)
Age group (years)	60–69	141 (46.2)
	70–79	107 (35.1)
	80–89	52 (17.1)
	≥90	5 (1.6)
Marital status	Married	233 (76.4)
	Widowed	46 (15.1)
	Single/Separated	26 (8.5)
Occupation	Retired	104 (34.1)
	Still employed (full/part-time)	88 (28.9)
	Unemployed	76 (24.9)
	Self-employed	37 (12.1)
Education	No formal education	64 (21.0)
	Primary	86 (28.2)
	Secondary	105 (34.4)
	Degree/Postgraduate	50 (16.4)
Socio-economic status (self-reported)	Low	70 (23.0)
	Middle	196 (64.3)
	High	39 (12.8)
Self-rated health	Excellent/Good	157 (51.5)
	Fair	114 (37.4)
	Poor	34 (11.1)
Chronic illness present	Yes	255 (83.6)
Annual health check-up	Yes	205 (67.2)
Financial satisfaction	Yes	216 (70.8)
Pension plan	Yes	195 (63.9)
Health insurance	Yes	172 (56.4)
Financial dependency	Fully self-reliant	147 (48.2)
	Partially dependent	105 (34.4)
	Fully dependent	53 (17.4)
Retirement planning (extensive)	Yes	107 (35.1)
Perceived financial security	Yes	188 (61.6)
Strong social support	Yes	164 (53.8)
Mentally prepared for ageing	Yes	208 (68.2)
Legal arrangements completed	Yes	136 (44.6)

Overall, 68.2% reported being mentally prepared for ageing. Bivariate analysis (Table 2) demonstrated that higher education, better self-rated health, annual health check-ups, pension coverage, financial security, retirement planning, and strong social support were significantly associated with preparedness.

**Table 2:** Bivariate associations with mental preparedness for ageing (N = 305)

Variable	Association with preparedness	p-value
Marital status	Married > single/widowed	0.004*
Occupation	Retired > unemployed/self-employed	0.035*
Education	Higher education → greater preparedness	0.005*
Health status	Better self-rated health → greater preparedness	0.008*
Annual health check-up	Yes > no/occasional	0.01*
Financial satisfaction	Satisfied > not satisfied/unsure	0.03*
Pension plan	Present > absent	0.019*
Financial dependency	Self-reliant > dependent	0.017*
Retirement planning	Yes (extensive) > none	0.01*
Perceived financial security	Yes > no/unsure	<0.01*
Reflection on ageing	Frequent reflection > none/occasional	0.011*
Post-retirement planning	Yes > no	0.011*
Social support	Strong > isolated/weak	0.0004*

In the multivariate model (Table 3), four independent predictors emerged: absence of a pension (adjusted odds ratio (AOR) = 2.24, p = 0.014), lack of health insurance (AOR = 0.36, p = 0.003), financial insecurity (AOR = 4.08, p = 0.0004), and weak or absent social support (AOR = 7.56, p = 0.0003). Other variables, such as education, self-rated health, and retirement planning, were not retained after adjustment.

**Table 3:** Multivariate ordinal logistic regression predicting mental preparedness (N = 305)

Predictor	Adjusted odds ratio (95% CI)	p-value
No pension plan	2.24 (1.18 – 4.24)	0.014*
No health insurance	0.36 (0.19 – 0.71)	0.003*
Financial insecurity	4.08 (1.88 – 8.85)	0.0004*
Unsure about financial security	3.95 (1.82 – 8.56)	0.0005*
Weak/isolated social support	7.56 (2.51 – 22.80)	0.0003*

## Discussion

This pilot hospital-based study assessed preparedness for ageing among older adults in Kannur, Kerala. Approximately two-thirds of participants (68.2%) reported being mentally prepared for ageing. Preparedness was positively associated with financial security, pension coverage, health insurance, and strong social support. In contrast, legal preparedness was low, with fewer than half of participants having wills or power of attorney. These findings underscore that financial and social domains are key drivers of perceived readiness, whereas legal and long-term planning remain underdeveloped.

The prevalence of preparedness observed here is higher than reports from other Indian settings, where mental health vulnerabilities and low planning for ageing are common [1,2]. For instance, the India Ageing Report 2023 highlights widespread psychological distress and weak planning among older adults, particularly in urbanising regions [1]. The strong association between social support and preparedness in our study is consistent with international evidence that community ties and family support improve well-being in later life [10]. Similarly, the link between education and preparedness aligns with global studies showing that education enhances access to information and financial literacy [11,12].

Despite Kerala’s high literacy and health indices, gaps in financial and legal preparedness persist. [13] This paradox may reflect overreliance on family-based support systems, cultural reluctance to formalise legal arrangements, and limited financial counselling targeted at the elderly. The finding that many participants reported satisfaction with savings but had not engaged in structured retirement planning suggests a potential overestimation of preparedness or lack of financial literacy, a phenomenon also observed internationally [5]. The high prevalence of chronic illnesses, despite frequent health check-ups, points to the limitations of current geriatric care services in addressing multimorbidity.

These findings highlight the need for integrated interventions. Policies must go beyond improving literacy and health access, focusing instead on (i) strengthening financial education and retirement planning programmes, (ii) expanding pension and insurance coverage, (iii) promoting legal literacy through community awareness campaigns, and (iv) embedding ageing preparedness counselling into routine healthcare. Kerala’s strong social development history makes it a suitable setting to pilot such reforms, which are aligned with the United Nations Population Fund's (UNFPA) call for “ageing-in-place” policies and the WHO’s Age-Friendly City framework [14].

Limitations

This study has several important limitations. First, its cross-sectional, hospital-based pilot design with purposive sampling restricts generalisability, as participants may differ from community-dwelling older adults. Second, mental preparedness was assessed using a single, self-developed item, which underwent expert validation but was not formally psychometrically validated or reliability-tested, limiting construct validity. Third, although data collection was performed by trained personnel with standardised instructions, inter-rater reliability was not assessed, and interviewer bias cannot be ruled out. Fourth, recruitment occurred over a short three-month period (March-May), which may not reflect seasonal variations in mental well-being. Finally, unmeasured confounders such as diet, functional status, and psychological health were not captured. These limitations highlight the exploratory nature of this pilot study, and findings should be interpreted as preliminary associations rather than definitive or generalisable conclusions.

Strengths

Despite its limitations, this study has several notable strengths. To our knowledge, it is among the first pilot studies in India to focus specifically on the underexplored concept of mental preparedness for ageing. By incorporating multiple domains, financial, health, social, and legal, the questionnaire provided a multidimensional perspective rather than focusing solely on physical or medical aspects. The relatively large sample size for an exploratory pilot (n = 305) enhances the robustness of preliminary analyses. Furthermore, the use of multivariate ordinal regression allowed identification of independent associations, strengthening the analytic rigour compared to purely descriptive reports. Finally, by situating the study in Kerala, a state with advanced ageing demographics but paradoxical gaps in financial and legal preparedness-this research adds contextually relevant evidence that can inform future community-based and longitudinal investigations.

## Conclusions

This pilot hospital-based study provides early insights into the mental preparedness of older adults in Kerala, highlighting strong associations with financial security, pension coverage, health insurance, and social support. While the findings underscore important gaps in legal and long-term planning, they must be interpreted with caution due to the use of a single, non-validated item to measure preparedness. The novelty of the study lies in its focus on mental preparedness, a dimension rarely explored in India, and its multi-domain approach spanning financial, health, social, and legal aspects. These exploratory results suggest that interventions promoting financial literacy, pension coverage, insurance enrolment, and social connectedness may improve preparedness for ageing. However, future community-based, longitudinal studies employing validated instruments are essential to confirm these associations, address unmeasured confounders, and provide more generalisable evidence to guide policy and practice.

## Appendices

Appendix A 

**Table 4:** Preparing for ageing questionnaire

Section	Item No.	Question	Response Options
1. Demographics	1	Age	_______
	2	Sex	☐ Male ☐ Female ☐ Other ☐ Prefer not to say
	3	Marital status	☐ Single ☐ Married ☐ Widowed ☐ Divorced ☐ Separated
	4	Current occupation status	☐ Retired ☐ Working full-time ☐ Working part-time ☐ Self-employed ☐ Unemployed
	5	Highest level of education	☐ No formal education ☐ Primary ☐ Secondary ☐ College/University ☐ Postgraduate
	6	Socioeconomic status (self-reported)	☐ Low ☐ Middle ☐ High
2. Health & well-being	7	Current health status	☐ Excellent ☐ Good ☐ Fair ☐ Poor
	8	Presence of chronic illness	☐ Yes ☐ No
	9	Frequency of health check-ups	☐ Yes, annually ☐ Yes, occasionally ☐ No
3. Financial preparedness	10	Satisfied with financial savings?	☐ Yes ☐ No ☐ Not sure
	11	Do you have a pension plan?	☐ Yes ☐ No
	12	Do you have health insurance?	☐ Yes ☐ No
	13	Financial dependency	☐ Fully self-reliant ☐ Partially dependent on spouse/children ☐ Fully dependent on spouse/children
	14	Retirement financial planning	☐ Yes, extensively ☐ Yes, somewhat ☐ No, not at all
	15	Do you feel financially secure?	☐ Yes ☐ No ☐ Unsure
4. Mental & emotional preparedness	16	Thoughts about ageing	☐ Yes, frequently ☐ Occasionally ☐ Never
	17	Planning for post-retirement life	☐ Yes, extensively ☐ Yes, somewhat ☐ No, not at all
	18	Plans after retirement (multiple select)	☐ Hobbies ☐ Traveling ☐ Volunteering ☐ Time with family ☐ Learning new skills ☐ Other: _______
	19	Social support system	☐ Yes, very strong ☐ Somewhat ☐ No, I feel isolated
	20	Mentally prepared for ageing	☐ Yes ☐ No ☐ Not sure
5. Future planning & preferences	21	Future living arrangement	☐ Stay in own home ☐ Move in with family ☐ Assisted living/retirement home ☐ Not thought yet
	22	Legal arrangements (wills, power of attorney)	☐ Yes, fully prepared ☐ Partially prepared ☐ No, not yet
	23	Biggest concerns about ageing (multiple select)	☐ Health decline ☐ Financial insecurity ☐ Losing independence ☐ Loneliness/isolation ☐ Becoming a burden ☐ Other: _______

## References

[1] United Nations Population Fund (UNFPA) and International Institute for Population Sciences (IIPS) (2025). UNFPA: Caring for Our Elders Institutional Responses. 'INDIA AGEING REPORT 2023'. UNFPA: Caring for Our Elders Institutional Responses, 'India Ageing Report 2023'.

[2] International Institute for Population Sciences (IIPS); National Programme for Health Care of the Elderly (NPHCE), Ministry of Health & Family Welfare, Government of India (2025). Longitudinal Ageing Study in India (LASI) Wave 1 Report: An Investigation of Health, Economic, and Social Well‑being of India’s Growing Elderly Population. Longitudinal Ageing Study in India (LASI) Wave 1 Report: An Investigation of Health, Economic, and Social Well‑being of India’s Growing Elderly Population.

[3] Solhi M, Pirouzeh R, Zanjari N, Janani L (2022). Dimensions of preparation for aging: a systematic review. Med J Islam Repub Iran.

[4] Prince MJ, Wu F, Guo Y, Gutierrez Robledo LM, O'Donnell M, Sullivan R, Yusuf S (2015). The burden of disease in older people and implications for health policy and practice. Lancet.

[5] Chen A, Yin Y, Munnell AH (2025). How well do people perceive their retirement preparedness?. Center for Retirement Research at Boston College.

[6] Noone JH, Stephens C, Alpass FM (2009). Preretirement planning and well-being in later life: a prospective study. Res Aging.

[7] Hershey DA, Henkens K, Van Dalen HP (2010). Aging and financial planning for retirement: interdisciplinary influences viewed through a cross-cultural lens. Int J Aging Hum Dev.

[8] Rajan SI, Shajan A, Sunitha S (2020). Ageing and elderly care in Kerala. China Rep.

[9] Cunha SD, Suresh S, Yathindra C (2019). Rights of the elderly: awareness study among elderly. Int J Health Sci Res.

[10] Holt-Lunstad J, Smith TB, Baker M, Harris T, Stephenson D (2015). Loneliness and social isolation as risk factors for mortality: a meta-analytic review. Perspect Psychol Sci.

[11] (2025). World report on ageing and health. https://iris.who.int/bitstream/handle/10665/186468/WHO_FWC_ALC_15.01_eng.pdf.

[12] Belo P, Navarro-Pardo E, Pocinho R, Carrana P, Margarido C (2020). Relationship between mental health and the education level in elderly people: mediation of leisure attitude. Front Psychol.

[13] Trupti T, Sarkar M, Tanwar R, Kumari N (2023). A study to assess the awareness on rights of elderly among senior citizens at selected setting, Chennai, Tamil Nadu. Ind J Psychol.

[14] World Health Organization (2025). National programmes for age-friendly cities and communities A guide. National Programmes for Age-Friendly Cities and Communities: A Guide.

